# Novel and Emerging LDL-C Lowering Strategies: A New Era of Dyslipidemia Management

**DOI:** 10.3390/jcm13051251

**Published:** 2024-02-22

**Authors:** Federica Agnello, Salvatore Ingala, Giulia Laterra, Lorenzo Scalia, Marco Barbanti

**Affiliations:** 1 Division of Cardiology, Ospedale Umberto I, ASP 4 di Enna, 94100 Enna, Italy; federicagiuseppa.agnello@gmail.com (F.A.); ingalasalvatore@gmail.com (S.I.); lorenzoscalia1993@gmail.com (L.S.); 2 Faculty of Medicine and Surgery, Università degli Studi di Enna “Kore”, 94100 Enna, Italy

**Keywords:** dyslipidemia, LDL-C, ACS, statin, PCSK9i, bempedoic acid

## Abstract

Atherosclerotic cardiovascular disease (ASCVD) represents a major global health challenge, significantly contributing to mortality rates. This chronic inflammatory condition affecting blood vessels is intricately linked to hypercholesterolemia, with elevated levels of low-density lipoprotein cholesterol (LDL-C) recognized as a central and modifiable risk factor. The effectiveness of lipid-lowering therapy (LLT) in mitigating ASCVD risk is well established, with studies revealing a substantial reduction in major ischemic events correlating with LDL-C reduction. While statins, often combined with ezetimibe, remain fundamental in dyslipidemia management, a significant proportion of patients on statin therapy continue to experience cardiovascular events. Recent pharmacological advancements, driven by a deeper understanding of atherogenesis, have unveiled novel therapeutic targets and potent drugs. Notably, agents like bempedoic acid and proprotein convertase subtilisin/kexin type 9 (PCSK9) inhibitors (evolocumab, alirocumab, inclisiran) have emerged as effective options to intensify LLT and achieve LDL-C goals, addressing limitations associated with statins, such as myopathy. Molecular insights into alternative pathways have spurred the investigation of emerging agents, offering promising perspectives for novel medications with efficacy comparable to established treatments, associated with advantages in cost and administration. This review provides a comprehensive overview of the evolving landscape of lipid-lowering strategies, highlighting the progress made in addressing ASCVD risk and the potential of upcoming therapies to further optimize cardiovascular prevention.

## 1. Introduction

Atherosclerotic cardiovascular disease (ASCVD) is a chronic inflammatory disease affecting blood vessels, and it stands as a leading cause of mortality [1]. The development and progression of ASCVD involve many determinants, with hypercholesterolemia playing a central role. Specifically, elevated levels of low-density lipoprotein cholesterol (LDL-C) have historically been identified as the pivotal target, that is due to its tightly associated extent and duration of exposure with the risk of ASCVD in all of its manifestations [2,3]. Studies about lipid-lowering therapy (LLT) revealed a decrease of major ischemic events by 40–50% for each LDL-C reduction of 30 mg/dL [4,5,6]. Therefore, great efforts in cardiovascular prevention are focused to reduce LDL-C levels, encouraging lifestyle interventions and implementing medications.

Statins, often combined with ezetimibe, constitute the cornerstone in dyslipidemia management and remain the most commonly prescribed lipid-lowering agents. However, despite significant improvements in cardiovascular outcomes, up to 40% of patients on statin therapy continue to suffer from cardiovascular events [7,8]. Pharmacological advancement, coupled with a deeper understanding of atherogenesis, has led to the identification of novel therapeutic targets and the development of new potent drugs, that are now available, namely, the proprotein convertase subtilisin/kexin type 9 (PCSK9) inhibitors (evolocumab, alirocumab, inclisiran) and the bempedoic acid. These drugs are effective in intensifying LLT and reaching LDL-C goals and dealing with issues associated with statins, such as myopathy (Table 1). Furthermore, molecular improvement found other pathways and new strategies to target it; thus, many emerging agents are currently under investigation, promising the opportunity of novel medications as powerful as the established ones, with the convenient cost of production and comfortable route of administration (Table 2).

Alongside pharmacological treatment, another noteworthy therapeutic option is plasmapheresis, which still plays a significant role in homozygous familial hypercholesterolemia. This procedure consists of removing and filtering a patient’s plasma to reduce cholesterol levels, offering an alternative approach to managing this particular form of hypercholesterolemia, particularly when the pharmacological LLT fails in achieving the target.

This review shows the landscape of the novel and the emerging LDL-C-reducing therapies and their mechanisms, discusses their future perspective, and offers a glimpse of the current era in lipidology, with a paradigm shift to a stricter and earlier lipid control. Moreover, the manuscript provides practical insights by suggesting an algorithm for optimal management of LLT in patients with acute coronary syndrome (ACS), representing actionable guidance for physicians.

## 2. Current Recommendation

The study of atherosclerosis and the strategies to control ASCVD emphasize the importance of reducing LDL-C through appropriate lifestyle interventions and lipid-lowering drugs. The intensity of recommendations and treatment goals are undoubtedly tailored to individual cardiovascular risk, ranging from dietary advice and physical activity for low-risk patients to aggressive pharmacological treatment in cases of established ASCVD [9]. In particular, the 2019 European Society of Cardiology (ESC) and European Atherosclerosis Society (EAS) guidelines focus on the presence of clinically or imaging-documented ASCVD, diabetes mellitus, severe renal disease, or one high uncontrolled risk factor. Patients with these conditions are defined as at high or even very high risk according to the guidelines [10]. For apparently healthy subjects aged ≥40 years, the 2019 ESC guidelines recommend calculating the risk onset of the first ASCVD manifestation at ten years using the systematic coronary risk estimation (SCORE)2 system, which incorporates the combined effect of different risk factors. Compared to the past, the guidelines emphasize a dynamic concept that considers the exposure time of a given risk factor or a possible combination. Consequently, individuals are stratified into low, moderate, or high risk, based on the calculated SCORE2, which is similar to the model proposed in the 2018 AHA/ACC/multi-society blood cholesterol management guidelines [11,12].

**Figure 1:**
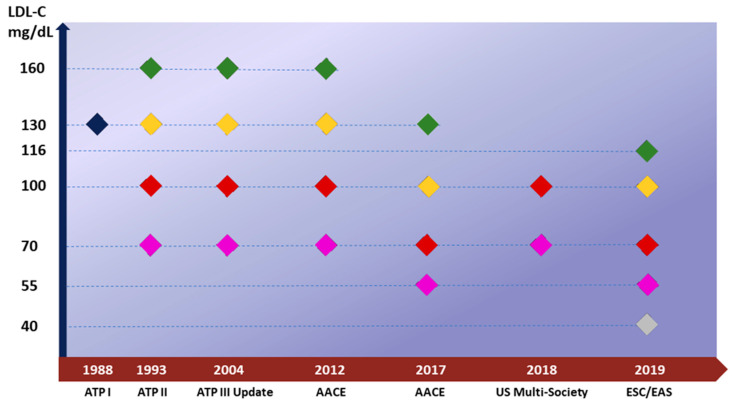
LDL-C goals evolution across guidelines over time. The timeline illustrates the evolution of LDL-C targets over time, reporting the recommended LDL-C goal across the different guidelines. The Adult Treatment Panel I (ATP I) guidelines in 1988 first deemed a desirable LDL-C target of 130 for all individuals at risk of ASCVD in primary prevention. Afterward, specific LDL-C cutoffs were established for secondary and primary prevention based on risk categories. These goals have become progressively stringent over time, culminating in the 2019 ESC guidelines, which underline a particularly lower LDL-C goal of 40 mg/dL for patients with repeated events within 2 years (silver marker). In the figure, the color marker represents the class of ASCVD risk (green for low risk, yellow for moderate risk, red for high risk and violet for very high risk respectively), while the very high risk is indicated in the box. The ATP I outlined an LDL-C target for all individuals (blue marker), independently of risk categories. Abbreviations: AACE, American Association of Clinical Endocrinologists; ATP, Adult Treatment Panel; EAS, European Atherosclerosis Society; ESC, European Society of Cardiology; LDL-C, low-density lipoprotein cholesterol.

Furthermore, both the 2019 ESC/EAS and the 2018 AHA/ACC/multi-society guidelines list some risk enhancers, such as chronic inflammatory disease or obesity, to consider in global risk assessment. Robust lines of evidence from outcomes trials suggest that lowering LDL-C is linearly associated with fewer ASCVD events. Thus, over time, the trend has evolved toward lower LDL-C goals, especially in secondary prevention, until reaching the ones outlined by the 2019 ESC/EAS guidelines. These recommend the following: for people at high cardiovascular risk, a reduction of LDL-C ≥ 50% from the baseline and a target LDL-C < 70 mg/dL; for very-high risk patients, a reduction of LDL-C ≥ 50% compared with the baseline and a target LDL-C < 55 mg/dL (Figure 1) [10,13,14].

Indeed, the authors of the guidelines set a stricter LDL-C goal of < 40 mg/dL, for individuals who experienced more than one event within 2 years, despite maximally tolerated statin treatment. Remarkably, in secondary prevention, the priority is to immediately reduce the exposure to LDL-C, aiming at atherosclerotic plaque stabilization and regression [15].

Such low LDL-C goals, practically, rule out statin monotherapy. Therefore, a stepwise approach is recommended for apparently healthy people at high or very high risk and for patients with established ASCVD, diabetes mellitus type 2, chronic kidney disease, or familial hypercholesterolemia (class I). This includes lifestyle measures (class I), followed by the addition of PCSK9 inhibitors (PCSK9i) in case of failure to achieve the LDL-C goal on high-intensity statin therapy at the maximum tolerated dose (class I) and ezetimibe (class I) [10,16]. Moreover, these drugs are primarily recommended in the context of combination therapy with statins and ezetimibe as a strategy for secondary prevention of cardiovascular events (class I); however, they are also recommended in the context of primary prevention in patients at very high risk with familial hypercholesterolemia (class I) or without such risk (class IIb). PCSK9i in combination with ezetimibe also represents a statin alternative in case of intolerance (class IIb) [10,16,17]. Future ESC guidelines on dyslipidemia management will include, among non-statin therapies, the newer lipid-lowering agents, which received approval after the last published guidelines (evinacumab, inclisiran, bempedoic acid).

## 3. Consolidated Approaches: Certainty and Weakness

As previously mentioned, the realm of therapeutic armamentarium for lipid disorders has further expanded over the past few years. Nonetheless, the pillars of LLT currently remain the most studied, with extensively documented efficacy and well-known strengths and limitations.

Statins are the milestone of lipid control. They inhibit 3-hydroxy-3-methylglutaryl-coensyma A reductase, resulting in lower intrahepatic cholesterol and an upregulation of LDL receptors (LDLR) in hepatic cells, which finally enhances the uptake of circulating LDL-C. The magnitude of LDL-C reduction on statin therapy depends on the dose, type, and individual response, allowing a reduction of ≥50% with a high-intensity statin regimen and, indeed, a significant reduction in major adverse cardiovascular events [4,10,11]. Notably, statin therapy has shown a decrease in lipid-rich plaque and an increase in calcification in studies using intravascular ultrasonography or coronary computed tomography angiography [18,19]. Although statins are usually well tolerated, 15% of patients complain of muscle symptoms during treatment, which is the most frequent adverse event of the class. Only a minority of individuals develop myopathy—a large part of these perceived symptoms is associated with the nocebo effect, but it plays a crucial role in statin discontinuation and medical nonadherence [20]. Furthermore, statins could affect glucose homeostasis and increase the risk of new-onset diabetes, which has been related more to rosuvastatin compared with atorvastatin in a recent trial [4,10,21].

Ezetimibe reduces intestinal absorption of cholesterol, targeting the Niemann–Pick C1-like 1 protein [22]. Many studies demonstrated that ezetimibe added to statin produces an additional 24% LDL-C reduction compared with statin monotherapy, significantly reducing the recurrence of cardiovascular events. It is usually well tolerated; moreover, the numerous fixed-dose formulations with different statins contribute to improving medication adherence [23,24,25].

The two human monoclonal antibodies (mAbs) against PCSK9 currently available, evolocumab and alirocumab, have a central role in dyslipidemia management, especially after the ambitious LDL-C goals outlined in the last guidelines. A compelling body of evidence supports the efficacy of PCSK9i in reducing LDL-C levels by up to 60%, and even more when combined with other lipid-lowering drugs [17,26]. Furthermore, similarly to statins, intensive LLT with this class of agents has demonstrated its ability to reduce plaque burden and induce regression. This effect has been observed both in primary prevention among patients with familial hypercholesterolemia and in secondary prevention in patients with a previous cardiovascular event [27,28]. Both evolocumab and alirocumab have shown a favorable safety profile in large-scale clinical trials as well as in real-world studies, revealing mild side effects like injection-site reactions, nasopharyngitis, and upper respiratory infections. The main trials assessing the safety of these medications focused on the potential onset of neurocognitive alterations, cancer recurrence or worsening, new-onset diabetes, rhabdomyolysis, and death, but no significant difference emerged [29]. Compared with statins, PCSK9i mAbs provide a convenient dose frequency (monthly or bimonthly), enhancing patient adherence. However, they are expensive and this is a remarkable limit to their implementation in clinical practice.

## 4. Innovative Approaches and Investigational Therapies

In the evolving landscape of lipid management, a range of innovative strategies has emerged. Bempedoic acid, a unique ATP citrate lyase inhibitor, stands out as a promising fresh approach to cholesterol synthesis. PCSK9i, renowned for their impact on LDLR activity, and angiopoietin-like protein 3 (ANGPTL3) inhibitors, influencing lipid metabolism, bring novel dimensions to LDL-C reduction.

The revolutionary CRISPR/Cas9 technology offers a genetic editing marvel, directly targeting genes involved in cholesterol regulation. Concurrently, cholesteryl ester transfer protein (CETP) inhibitors aim to optimize lipid profiles, influencing HDL-C and LDL-C dynamics. Exploring the frontier of vaccines for LDL-C reduction adds an immunotherapeutic dimension to lipid management.

This chapter delves into these emerging strategies, exploring their mechanisms, clinical implications, and the potential to redefine the landscape of LLT.

### 4.1. Bempedoic Acid

Bempedoic acid represents a therapeutic option to reduce LDL-C, which can be used on a large scale with low costs and easy administration. It is an oral, once-daily, small molecule with a half-life of 15–24 h and it is responsible for the inhibition of adenosine triphosphate–citrate lyase (ACLY), a cytosolic enzyme upstream of HMG-CoA reductase [30]. Bempedoic acid is a prodrug rapidly converted to a coenzyme A derivate at the hepatic level by an endogenous liver very long-chain acyl-CoA synthetase-1 (ACSVL1). Since ACSVL1 is not present in the skeletal muscle, there is less risk of myalgia symptoms and myopathy compared with statins [31]. During the bempedoic acid development program, many clinical studies have consistently demonstrated that this drug at a dose of 180 mg, the only one available, is able to significantly reduce LDL-C levels. In the CLEAR (Cholesterol Lowering via Bempedoic, an ACL-Inhibiting Regimen) Wisdom trial, 779 patients with ASCVD, heterozygous familial hypercholesterolemia, or both, were randomized to bempedoic acid add-on statin or placebo, showing, at week 12, that bempedoic acid significantly lowered LDL-C by 15.1% (*p* < 0.001) [31]. The impact on clinical outcomes was revealed in the CLEAR Outcomes trial, in which bempedoic acid treatment, compared with placebo, decreased the risk of major cardiovascular events among statin-intolerant patients at high risk or with established ASCVD (11.7% versus 13.3%; *p* = 0.004) [32]. Analysis of aggregate data from clinical trials highlighted that, in the absence of other LLT, bempedoic acid was associated with a 24% reduction in LDL-C levels [33]. Indeed, compared with placebo, the combination of bempedoic acid with moderate or high-intensity statin produced a greater reduction in LDL-C levels [34]. When bempedoic acid was tested in fixed combination with ezetimibe, an even more remarkable effect was observed in terms of reduction in LDL-C levels (−38% compared with the baseline, placebo-corrected percentage change) [35]. The efficacy of bempedoic acid is accompanied by a favorable safety profile, without increasing new-onset diabetes and muscle disorder, whereas a modest but significantly higher risk of gout was observed compared with placebo [36,37].

This non-statin agent was approved in 2020 both by the European Medicines Agency (EMA) and by the Food and Drug Administration (FDA). According to the 2022 Expert Consensus Decision Pathway on the Role of Nonstatin Therapies for LDL-C Lowering, bempedoic acid is an additional therapy for patients with ASCVD at very high risk who do not achieve LDL-C goals despite maximally tolerated statin, ezetimibe, or an anti-PCSK9 mAbs therapy [38].

### 4.2. Novel Strategies for PCSK9 Inhibition

PCSK9 is a key element in LDL-C homeostasis as it promotes the degradation of LDLR on the liver surface, thus preventing the uptake of circulating LDL-C. Consequently, it has been targeted at various levels by many medications whose efficacy is based on intrinsic LDLR function [39]. Interest in the development of other PCSK9i particularly increased after the successful efficacy of evolocumab and alirocumab, leading to the recent advent of inclisiran in clinical practice, and continuous efforts have been invested in this dedicated research field. The goal is to develop further agents that offer alternative strategies for aggressively controlling dyslipidemia. The PCSK9 inhibition strategies mainly differ in frequency of dosing, decreasing from biweekly or monthly for protein-targeting interventions to twice per year with small interfering ribonucleic acid (siRNA), to once-in-a-lifetime DNA-targeting approaches. Longer efficacy duration may improve convenience and adherence.

#### 4.2.1. Gene Silencing

The improvement of RNA therapeutics also involved the field of dyslipidemia, providing approaches to regulating the expression of atherogenesis key players, just like PCSK9. That is the basis of gene silencing, which is a post-transcriptional process aiming at the temporary or permanent suppression of a specific gene’s activity, without altering its sequence. Currently, PCSK9 gene silencing is feasible through two approaches targeting the mRNA of the PCSK9 gene, including siRNA and antisense oligonucleotides (ASOs).

Inclisiran—It is a novel, subcutaneously administered, therapeutic first-in-class agent, which exerts its molecular effects through a siRNA unique mechanism, targeting the hepatic PCSK9 mRNA. Upon administration, inclisiran binds to the hepatic cells and specifically inhibits the expression of PCSK9 mRNA thanks to the Argonaute-2 protein. This interference disrupts the molecular cellular processes that lead to PCSK9 synthesis, which promotes LDLR recycling and their hepatocyte upregulation [40]. Similar to bempedoic acid, the approval of inclisiran for clinical use is recent, but a wealth of scientific evidence supports its prominent role.

The phase-2 ORION 1 trial randomized 501 patients at high risk for ASCVD to inclisiran at six different doses or to placebo: the findings indicated that LDL-C was cut by 27.9–41.9% with one subcutaneous inclisiran injection and by 35.5–52.6% with two injections (*p* < 0.001), with effect persisting over 240 days [41].

The ORION-10 and ORION-11 trials respectively enrolled 1561 and 1617 patients with ASCVD, who had elevated LDL-C levels despite statin therapy at a maximum tolerated dose and who were randomized to placebo or inclisiran (by subcutaneous injection on day 1, day 90, and every 6 months thereafter over a period of 540 days). At day 510, in both studies, inclisiran was associated with a significant decrease in LDL-C level, by 51.3% in the first trial (*p* < 0.001) and by 45.8% in the second one (*p* < 0.001) [42].

Some evidence of the long-term efficacy and safety of inclisiran is provided by the ORION 3, a 4-year open-label extension study of the phase 2 ORION-1 trial, in which inclisiran therapy resulted in sustained reductions in LDL-C and PCSK9 concentrations, with good tolerability [43].

Furthermore, this agent provides a more targeted and effective solution for genetic-based hypercholesterolemia, confirming its efficacy in reducing LDL-C both among patients with heterozygous and homozygous familial hypercholesterolemia already on guidelines-recommended therapy [44,45].

Although inclisiran is approved for LDL-C lowering, it has not yet been established whether this approach will result in a reduction in clinical events as has been shown with other therapies targeting PCSK9. Inclisiran is being tested in the ongoing ORION-4 (NCT03705234) and VICTORION-2 Prevent (NCT05030428) trials in this respect. These randomized studies will provide a wider number of patients exposed to inclisiran.

ASOs—Another strategy of gene silencing involves the use of ASOs, which could be administered subcutaneously and potentially orally. These synthetic single-stranded nucleotides are designed to complementarily bind the target mRNA, mimicking the complex DNA-RNA and activating cleavage by RNAsi H1. This process ultimately inhibits mRNA translation, specifically in the case of PCSK9 synthesis [46]. First-generation PCSK9 ASOs, such as ISIS-394814/BMS-844421 and SPC4061/SPC5001, exhibited limited affinity and safety issues, leading to the discontinuation of further investigation. Subsequently, advancement in ASO technology involved structural modifications to enhance binding affinity and target organ specificity while minimizing immunostimulatory activity, producing the development of a new generation of ASOs [47,48]. One of these is AZD8233 (ION-863633), an investigational ASO conjugated with an N-acetylgalactosamine (GalNAc) molecule, specifically uptaken by the liver where it targets PCSK9 gene expression at the nuclear level. In a phase I study (NCT03593785), subcutaneous administration of a single injection showed a reduction in plasma PCSK9 levels of up to 90% and a decrease in circulating LDL-C value by up to 68% over a month. In two phase 2b randomized trials, one monthly injection of AZD8233 showed a significant and robust reduction in LDL-C in high-risk hypercholesterolemia patients, but it did not achieve prespecified efficacy criteria set by the industry. Hence, the company has decided not to advance AZD8233 into phase 3 trials, and analysis of the results is still ongoing to outline the next steps [49]. Moreover, AZD8233 has oral potential, which could represent a convenient strategy in terms of patient comfort and therapeutic adherence, although tablet formulation currently has been tested only in animal models [50].

#### 4.2.2. Binding Peptides

This therapeutic class of drugs acts by binding PCSK9 and then preventing its interaction with LDLR. Many emerging agents, different in chemical structure, reduce LDL-C levels through this mechanism.

Adnectines—Adnectines are small inhibitory proteins subcutaneously or intravenously administered, which bind PCSK9 with high affinity. Preclinical and early clinical studies found promising results, particularly LIB003 showed effectiveness and tolerability in the phase II trial, therefore this molecule is currently under investigation in the phase 3 trial (NCT04797247).

Macrocyclic peptides—A relevant group of biding agents is represented by macrocyclic peptides, designed using mRNA display combined with unnatural amino acid residues. These molecules offer a novel approach with affinity and specificity comparable to mAbs, but at a lower cost of production and with oral availability. The macrocyclic peptide MK-0616 reduced over 93% in the geometric mean of free, unbound plasma PCSK9 in phase 1 clinical trials [51]. Recently, a phase 2b randomized, double-blind, placebo-controlled trial testing daily MK-0616 at various dosages (from 6 mg to 30 mg) showed significant dose-dependent LDL-C reduction at 8 weeks, without tolerability concerns [52]. The phase 3 CORALreef Lipids trial (NCT05952856) is ongoing and will assess the safety and efficacy among patients with hypercholesterolemia.

Mimetic peptides—Mimetic peptides are short amino acidic sequences developed to mimic the specific structure of the target protein. The epidermal growth factor (EGF)-like repeat A and EGF-AB binding domains of LDLR are the most emulated peptic structure to inhibit the interaction between LDLR and PCSK9 through this mechanism. Many of these competitive molecules showed effective inhibition of LDLR degradation in in vitro studies on hepatic cells, such as Pep2-8 mimicking the EGF-A catalytic domain. Similarly, a mimetic peptide binding the PCSK9 isolated C-terminal domain (H306Y) seems to be a promising peptide too. Therefore, mimetic peptides offer a promising novel strategy to inhibit PCSK9-mediated degradation of LDLR, allowing LDL-C uptake, but further research is necessary to assess their safety and clinical efficacy [53].

Small molecules—Opposite the large mAbs, small molecules could inhibit PCSK9 through different mechanisms, from the inhibition of PCSK9 and LDLR interaction to interference during the PCSK9 secretion process or synthesis [17]. Great efforts have been spent to find out structural pocket in PCSK9—for example, NYXPCSK9i operates at the submicromolar level leading to potent disruption of the PCSK9-LDLR interaction in in vitro studies. The oral administration in mice study showed a dose-dependent decrease in plasma total cholesterol of up to 57% and amplified effects in combination with atorvastatin. Another small molecule, called 7030B-C5, acts as a transcriptional PCSK9 inhibitor, which downregulates its expression, increasing LDLR plasmatic levels and function. This class of compounds has attracted attention, representing another alternative approach with a low cost of production and oral delivery. Their investigation is in a very early phase, and further research is needed to address development challenges, including plasmatic stability, and assess their clinical efficacy.

#### 4.2.3. Gene Editing

A cutting-edge therapeutic approach is represented by gene editing, consisting of the introduction of permanent mutation in the target gene. The current most intriguing gene editing technologies are based on the CRISP-Cas9 system, which is characterized by the ability to introduce mutations on a genomic site with high specificity. Particularly, VERVE-101 is a CRISP-Cas 9-based genome editing medication realized to permanently inactivate PCSK9 gene expression, which showed efficacy in reducing PCSK9 level and circulation LDL-C in nonhuman primates and mouse models [54]. The ongoing Heart-1 trial (NCT05398029) is the first-in-human study investigating a single intravenous infusion of VERVE-101 in patients with heterozygous familial hypercholesterolemia and established ASCVD, on maximally tolerated oral lipid-lowering therapy [55]. Early results demonstrate efficacy in PCSK9 down expression, with a mean reduction of 69% in LDL-C levels at the higher administered dosage, without safety concerns in the short term, providing proof of concept for in vivo DNA base editing in humans. Although these are very exciting data, this strategy introduces genomic mutations; therefore, considerable surveillance for long-term safety issues is of relevant importance. Moreover, the life-treating effect of VERVE-101 suggests that it could be a revolutionary medication ideal for young patients in the early phase of the disease.

#### 4.2.4. Vaccines

A vaccine to manage atherosclerosis has always been an attractive strategy. Among the tested vaccine approaches, there is one against PCSK9, aiming at inducing durable and highly specific PCSK9 antibodies to prevent its interaction with the LDLR [56,57,58,59]. Many PCSK9 vaccine formulations showed efficacy and safety profiles in preclinical studies. In particular, the epitope vaccine AT04A consists of amino acidic sequences derived from the N-terminal region of PCSK9 combined with a carrier protein containing T-cell epitopes. It demonstrated to trigger PCSK9 antibodies, significantly reducing elevated LDL-C levels, decreasing plasma inflammation markers, and reducing atherosclerotic lesions in mice. Likewise, the early results of a vaccine derived from a short amino acidic sequence of PCSK9 are promising [60].

Furthermore, another potential active immunization could be obtained using short PCSK9-derived peptides linked to bacteriophage virus-like particles [61]. The development of the ideal vaccine formulation is challenging because it should disrupt the body’s tolerance to self-antigens without triggering an immune response from autoreactive T-cells that could attack healthy cells. Thus, despite the promising early results, vaccine experimentation has never gone beyond animal studies.

### 4.3. ANGPTL3 Inhibitors

ANGPTL3 is a glycoprotein expressed by the hepatic cells and secreted into circulation, which regulates lipid metabolism through the inhibition of lipoprotein and endothelial lipases. ANGPTL3 loss-of-function carriers have low levels of both LDL-C and triglycerides, with lower ASCVD risk. These observations paved the way for the development of a novel lipid-lowering class of drugs inhibiting ANGPTL3 with different strategies, including mAbs, ASOs, and siRNA, similar to PCSK9 inhibitors but reducing cholesterol with an LDLR-independent mechanism.

ANGPTL3 mAbs—Evinacumab is a fully human mAb against ANGPTL3, manufactured using Regenero’s VelocImmune technology, which relies on genetically engineered mice with a genetically humanized immune system, capable of producing fully human antibodies.

Evinacumab is administrated subcutaneously or intravenously, and it binds ANGPTL3, preventing lipids hydrolysis. Early investigations showed that evinacumab was effective in reducing LDL-C and triglyceride concentrations in healthy individuals, with no evidence of serious adverse events. A phase II trial conducted in 9 adults with homozygous familial hypercholesterolemia, on maximum of tolerated LLT, showed the efficacy of evinacumab, with a mean reduction in LDL-C levels by 49% after 4 weeks [62]. Another phase II trial randomized 272 patients with refractory hypercholesterolemia, with or without heterozygous familial hypercholesterolemia, to evinacumab (subcutaneously or intravenously) or placebo, showing that evinacumab significantly reduced LDL-C levels by more than 50% at the maximum dose [63]. Long-term efficacy and safety, until 72 weeks, were demonstrated in a study extension [64].

The phase III ELIPSE HoFH trial randomized 65 patients with homozygous familial hypercholesterolemia to evinacumab (15 mg/kg every 4 weeks) or matched placebo. At 24 weeks, evinacumab was associated with a significant LDL-C reduction of 47.1%, compared with the increase observed in the placebo group, with similar rates of adverse events [65]. Computed tomography angiographies were performed in two patients of this population, before randomization and 6 months after, suggesting the potentiality for effective plaque regression. In addition, an open-label extension study supported these results, finding that LDL-C reduction was maintained throughout the 48-week extension [66].

In light of the ELIPSE HoF trial, in 2021, the FDA and the EMA approved evinacumab as adjunctive LLT in patients with homozygous familial hypercholesterolemia aged ≥12 years. Later, the FDA approved the same indication for younger patients aged 5–11 years.

Regarding this age group, a pivotal 3-part, single-arm trial investigated the use of evinacumab in this patient population. Particularly, part A is a phase 1b study assessing the safety and tolerability of the drug, while part B is a phase III study, which reported that evinacumab decreased LDL-C levels, with durable effects, through 24 weeks [67].

A single-arm, phase III study assessing the long-term safety and efficacy of evinacumab among 116 patients with homozygous familial hypercholesterolemia has completed the enrollment, although the results are not available yet (NCT03409744).

Hence, evinacumab represents a valid lipid-lowering strategy, particularly in homozygous familial hypercholesterolemia, reducing LDL-C upstream, independently of LDLR. However, the clinical impact and long-term safety need to be investigated in larger studies and broader populations.

ASOs—ANGPTL3-LRx, also known as vupanorsen, is a second-generation GalNAc3-modified ASO targeting hepatic ANGPTL3 mRNA, preventing its translation into protein. Early phase I and phase II trials revealed that subcutaneous administration significantly improved lipid profile, mainly through triglycerides reduction and with a modest impact on LDL-C [68,69]. Nonetheless, results from the TRANSLATE-TIMI 70 trial (Effect of Vupanorsen on Non-high-density Lipoprotein Cholesterol Levels in Statin-Treated Patients with Elevated Cholesterol) stopped further development of the agent because vupanorsen led to relevant elevation in hepatic fat and liver enzymes [70]. The industry discontinued the development program of vupanersen since the risks were not balanced by the benefits [71].

ANGPTL3 siRNA—RNA-based therapy approaches have been developed to target ANGPTL3, as in the case of ARO-ANG3, which is a siRNA. Early-stage clinical trials indicated that siRNA is safe, well tolerated, and effective, both in patients with and without familial hypercholesterolemia [72]. The ARCHES-2 (Study of ARO-ANG3 in Adults with Mixed Dyslipidemia) phase II b study demonstrated that ARO-ANG3 significantly decreased ANGPTL3 expression and atherogenic triglyceride-rich lipoproteins, LDL-C and total apoB, in patients with mixed dyslipidemia [73]. The Gateway (Study of ARO-ANG3 in Participants with Homozygous Familial Hypercholesterolemia) trial (NCT05217667) is an ongoing phase II study, assessing the efficacy and safety of ARO-ANG3 among patients with homozygous familial hypercholesterolemia. Currently, two other siRNAs target the ANGPTL3 gene. LY3561774 and ANGsiR10 are in the developmental pipeline, but they are still in the preclinical phase.

### 4.4. CETP Inhibitors

The CETP is a liver-synthesized glycoprotein, involved in the exchange of cholesteryl esters and triglycerides between high-density lipoproteins (HDL) and LDL or very low-density lipoprotein particles. The observation that loss of function mutations in the CETP gene led to markedly increased HDL-C plasma levels as well as reduced LDL-C plasma levels sparked interest in pharmacological inhibition of CETP [74,75]. Originally, the CETP inhibitors were developed aiming at increasing HDL-C to reduce the risk of cardiovascular events; none of the CETP inhibitors investigated received market authorization. In fact, the phase III trial of torcetrapib, the first CETP inhibitor, was disappointing, showing an increased risk of death [76,77,78]. Later, the other two CETP inhibitors, evacetrapin and dalcetrapib, were evaluated in phase III trials but showed to be largely ineffective. Conversely, anacetrapib added to statin therapy was shown to reduce the risk of MACE but only modestly; therefore, the drug development was halted [79,80,81].

Obicetrapib is an investigational, selective CETP inhibitor undergoing clinical development to reduce both LDL-C and the incidence of MACE. Recently, a phase II trial showed obicetrapib 10 mg, added to ezetimibe, and high-intensity statin decreased LDL-C by 55.6% [82]. Therefore, this drug is the most potent CETP inhibitor to date, which renewed the interest in this class of agents.

## 5. Moving to Intensive LDL-C Management

Historical concerns with very low LDL-C and cellular processes (synthesis of cell membranes, steroid hormones, and bile acids) have been disproved by the observation of a population occurring with low levels of LDL-C naturally or with genetic mutations [83]. Moreover, several clinical trials assessing lipid-lowering drug combinations have shown greater ASCVD risk reduction maintaining LDL-C levels < 25 mg/dL, without limiting safety concerns [84,85]. Noteworthy, the longer-term (up to 8.6 years) safety of very low LDL-C levels during evolocumab therapy comes from the FOURIER-OLE study (FOURIER Open-Label Extension), in which the primary end-point of adverse events incidence did not increase over time [86].

Furthermore, robust evidence based on randomized clinical trials and Mendelian randomization highlights the time sensitivity in decreasing LDL-C, particularly in secondary prevention [87].

**Figure 2:**
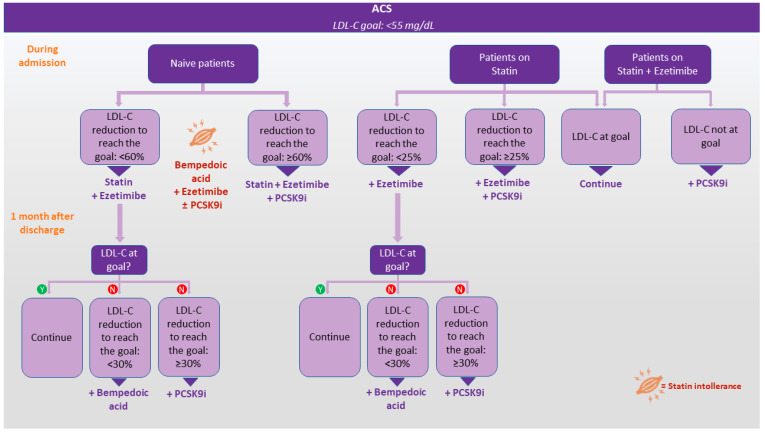
Proposed algorithm to manage LDL-C in patients with acute coronary syndrome. The figure illustrates the algorithm to control LDL-C in adult patients with ACS, as proposed by the authors. The therapeutic decision during hospitalization is guided by the presence or absence of prior LLT and the percentage reduction of LDL-C required to achieve the LDL-C goal of <55 mg/dL. At one-month reassessment, potential further optimization of therapy is indicated if the target has not been met. In the figure, “statin” refers to high-intensity statin therapy at the maximum tolerated dose. PCSK9i refers to one of the available drugs of this class (mAbs or siRNA, independently); thus, in case of intolerance or non-responsiveness, a switch to a different PCSK9i may be considered. The orange color, near the symbol indicating statin intolerance, highlights alternative therapeutic strategies to statins. The combination of these alternatives is guided by considering the LDL-C values at the baseline and the necessary percentage reduction to reach the target. The algorithm ensures a personalized approach to LLT, considering the patient’s prior treatment history and the individualized goal for LDL-C reduction. Abbreviations: ACS, acute coronary syndrome; LDL-C, low-density lipoprotein cholesterol; N = not; PCSK9i, proprotein convertase subtilisin/kexin type 9 inhibitors; Y = yes.

The significant strides in the field of dyslipidemia treatment provided us with a vast arsenal of powerful medications to strictly control lipid levels, in particular in very high-risk patients. Nevertheless, only a small percentage of this group achieves the LDL-C goal as emerged in a recent observational prospective study conducted in 14 European countries, which reported underutilization of combination therapies despite the aforementioned recommendation of 2019 ESC/EAS guidelines [88]. Currently, we can estimate the achievable LDL-C value with a drug alone or in combination, based on the available data: an average percentage reduction in LDL-C levels of 60% with PCSK9 inhibitors therapy alone, 75% in combination with high-intensity statins, and even 85% in combination with statins and ezetimibe [16]. Moreover, doubling the statin dose usually results in a further 6% reduction; thus, there is a paradigm shift toward “high-intensity LLT combination” as the first line, especially in very high-risk patients, which could be a pragmatic and effective approach [89]. Generally, LLT among naïve ACS patients may start with a combination of a statin and ezetimibe. In the case of statin intolerance, the combination could be represented by ezetimibe plus bempedoic acid or PCSK9i, according to the baseline lipid profile. A more intensive regimen including both the statin and ezetimibe and PCSK9 inhibitors should be prescribed to ACS patients already on a statin and ezetimibe therapy, as well as those ACS naïve patients whose LDL-C level should decrease ~80% to reach the LDL-C recommended level. Triple therapy may be also indicated for extremely high-risk patients. These authors propose an algorithm to establish LLT in adult patients with ACS, taking into account the baseline value of LDL-C, the presence of a previous LLT, and the LDL-C percentual reduction needed to reach the goal (Figure 2).

Sporadically, cases of reduced responsiveness to PCSK9i have been reported, defined as an LDL-C reduction of less than 15%. The causes could be different, such as decreased entry or absorption of PCSK9i, poor therapeutic adherence, development of neutralizing antibodies against PCSK9i mAbs, or discontinuation of the other lipid-lowering agents. The production of neutralizing antibodies is very rare and could potentially be mitigated by switching from PCSK9i mAbs to inclisiran. Therefore, an apparent resistance to PCSK9i often masks nonadherence to the complete LLT as prescribed. Nonetheless, bempedoic acid consistently represents a viable alternative in cases of intolerance or failure to achieve target levels, although it is not among the most potent available agents. However, there are no current data about a quadruple lipid-lowering association (statin, ezetimibe, PCSK9i, and bempedoic acid).

The gaps in the achievement of guideline-recommended LDL-C targets rely on different levels, including patient, physician, and regulatory factors. Among the other, there’s medication nonadherence, which is a well-known challenge in the cardiovascular field. Regarding LLT, some causes of nonadherence are represented by statin-related muscular symptoms, which could be solved using alternative agents if the intolerance is confirmed. In addition, the chronic duration of therapy and the pill burden of these patients are determinants. The use of polypill is an effective method to overcome the barrier of medication nonadherence [90]. Moreover, the development of current lipid-lowering agents is evolving toward less frequent administration, moving from daily oral tablets, to injectable agents administered monthly or twice a year, and even to lifelong treatments, such as gene editing. That considerably enhances medication adherence.

Another barrier to reaching guideline LDL-C targets is the cost related to PCSK9 mAbs and siRNA, which could be overcome by selecting patients at very high risk and with the advent of oral inhibitors. Indeed, education, close follow-up, and telemedicine for rural areas are other strategies to improve lipid control in clinical practice.

## 6. Authors’ Opinion

PSCK9 is the ideal target for reducing cholesterol levels, regardless of whether hypercholesterolemia derives from a gain of function in the PCSK9 gene or not. Currently, intensive LLT is dominated by PCSK9i, among which the mAbs represent the earliest and most successful strategy, with extensive supporting literature. However, the development of siRNA targeting PCSK9 mRNA added another powerful and safe drug to this pharmacological class, with effects similar to the mAbs, but with a potential advantage in terms of medication adherence, considering the biannual administration of inclisiran, compared with the monthly administration of the mAbs. On the other side, a rare but reported concern related to the mAbs is the development of resistance, which is due to the formation of neutralizing antibodies against the anti-PCSK9 mAbs, probably based on inter-individual variability. Considering the availability of different compounds inhibiting PCSK9, with different mechanisms but comparable effects, these authors believe that, beyond refining the existing therapy and assessing the efficacy of new agents, a critical need in this scientific field is the identification of criteria to better select patients to whom PCSK9i mAbs, rather than inclisiran, should be prescribed and vice versa. Therefore, future clinical studies will be comparing PCSK9i mAb versus inclisiran in a head-to-head trial.

## 7. Conclusions

In conclusion, lipid management stands as one of the most extensively researched fields, with numerous ongoing trials contributing to our evolving understanding. The deepening insights into the intricate pathways and molecules involved have led to the identification of novel therapeutic targets. Coupled with technological advancements, the past few years have witnessed a significant expansion in the repertoire of available lipid-lowering drugs. In particular, the introduction of inclisiran, bempedoic acid, and evinacumab for familial hypercholesterolemia has diversified therapeutic options, allowing for a more personalized approach. Today, clinicians have a spectrum of therapeutic choices to tailor interventions and achieve LDL-C target levels, taking part in the current orientation of a personalized secondary prevention therapy according to the residual cardiovascular risk [91,92,93]. Moreover, the promising results from various drugs currently in development hold the potential to further enhance clinical practice, overcoming some limitations of existing therapies. The continuous exploration of innovative solutions underscores the dynamic nature of lipid control in cardiovascular health.

## Figures and Tables

**Table 1:** Established lipid-lowering agents.

Drug	Mechanism of Action	Administration	Strength	Weakness	Adverse Reaction
Statin	Inhibition of HMG-CoA reductase	Oral tablets, once per day	LDL-C reduction ≥ 50% Strong evidence for ASCVD risk reduction Available as generic Available in FDC and polypill	Low adherence Muscle toxicity Nocebo effect	Muscle toxicity (myalgia, myopathy, myositis, rhabdomyolysis), transaminase elevation with rare risk for liver toxicity, risk for new-onset diabetes
Ezetimibe	Cholesterol absorption inhibitor	Oral tablets, once per day	Moderate evidence of secondary prevention Well tolerated Available as generic Available in FDC	Modest LDL-C reduction	Arthralgia, diarrhea, URTI
Evolocumab Alirocumab	Humanized monoclonal antibody against PCSK9	Subcutaneous injection, once a month	LDL reduction ≈60% Strong evidence for ASCVD risk reduction	High cost Injection	Injection-site reactions, nasopharyngitis, influenza
Inclisiran	siRNA against PCK9 mRNA	Subcutaneous injection, once on six months	LDL-C reduction ≥50%	High cost Injection No ASCVD outcomes	Injection-site reactions, arthralgia, UTI, diarrhea, bronchitis, pain in extremity, dyspnea
Evinacumab	Humanized monoclonal antibody against ANGPTL3	Endovenous infusion, once a month	+Statins: 47% (currently only approved for HoFH)	High cost Injection	Flu-like symptoms, nasopharyngitis, dizziness, rhinorrhea, nausea
Bempedoic Acid	ACL inhibitor	Oral tablets, once per day	Well tolerated Available in fixed-dose combination	Modest LDL-C reduction	Hyperuricemia, gout, cholelithiasis, URTI, muscle spasms, back pain, abdominal pain, pain in extremity, bronchitis, anemia, elevated liver enzymes

Abbreviations: HMG-CoA, 3-hydroxy-3-methylglutaryl coenzyme A; PCSK9, proprotein convertase subtilisin/kexin type 9; siRNA, small-interfering ribonucleic acid; mRNA, messenger ribonucleic acid; ANGPTL3, angiopoietin-like 3; ACL, adenosine triphosphate citrate lyase; LDL-C, low-density lipoprotein cholesterol; ASCVD, atherosclerotic cardiovascular disease; FDC, fixed-dose combination; URTI, upper respiratory tract infections; UTI, urinary tract infections.

**Table 2:** Emerging LDL-lowering therapies under development.

Target	Strategy	Agent	Phase	LDL Reduction	Administration
PCSK9	ASO	AZD 8233	II	68%	Subcutaneous injection, once a month Oral potential
	Adnectine	LIB003	II	≥50%	Subcutaneous injection, once a month
	Cyclic peptide	MK-0616	II	60.9%	Oral tablets, once per day
	Small molecules	NYXPCSK9i	I	57% total cholesterol	Oral tablets, once per day
	Vaccine	epitope vaccine AT04A	I	13%	Subcutaneous injection, once a year
	CRISP-Cas 9	VERVE-101	I	60%	For life
ANGPTL3	ASO	AKCEA-ANGPTL3-RX	I–II	33%	Subcutaneous injection, once a month.
	siRNA	ARO-ANG3	I	42%	Subcutaneous injection, once a month
CETP	CETP inhibition	Obicetrapib	II	45%	Oral tablets, once per day

Abbreviations: ASO, antisense oligonucleotides; CETP, cholesteryl ester transfer protein; PCSK9, proprotein convertase subtilisin/kexin type 9; siRNA, small interfering ribonucleic acid.

## Data Availability

No new data were created or analyzed in this study. Data sharing is not applicable to this article.
